# Collagen peptide modified carboxymethyl cellulose as both antioxidant drug and carrier for drug delivery against retinal ischaemia/reperfusion injury

**DOI:** 10.1111/jcmm.13768

**Published:** 2018-07-20

**Authors:** Hua Mu, Yeqing Wang, Haiying Wei, Hong Lu, Zhuolei Feng, Hongmin Yu, Yue Xing, Haijing Wang

**Affiliations:** ^1^ Department of Ophthalmology the First Affiliated Hospital of Harbin Medical University Harbin Heilongjiang China

**Keywords:** antioxidant carrier, drug delivery, IL‐10, retinal ischaemia/reperfusion injury

## Abstract

Oxidative stress can cause injury in retinal endothelial cells. Carboxymethyl cellulose modified with collagen peptide (CMCC) is of a distinct antioxidant capacity and potentially a good drug carrier. In this study, the protective effects of CMCC against H_2_O_2_‐induced injury of primary retinal endothelial cells were investigated. In vitro, we demonstrated that CMCC significantly promoted viability of H_2_O_2_‐treated cells, efficiently restrained cellular reactive oxygen species (ROS) production and cell apoptosis. Then, the CMCC was employed as both drug and anti‐inflammatory drug carrier for treatment of retinal ischaemia/reperfusion (I/R) in rats. Animals were treated with CMCC or interleukin‐10‐loaded CMCC (IL‐10@CMCC), respectively. In comparisons, the IL‐10@CMCC treatment exhibited superior therapeutic effects, including better restoration of retinal structural thickness and less retinal apoptosis. Also, chemiluminescence demonstrated that transplantation of IL‐10@CMCC markedly reduced the retinal oxidative stress level compared with CMCC alone and potently recovered the activities of typical antioxidant enzymes, SOD and CAT. Therefore, it could be concluded that CMCC provides a promising platform to enhance the drug‐based therapy for I/R‐related retinal injury.

## 1 INTRODUCTION

Retinal ischaemia/reperfusion (I/R) injury was one of important factors responsible for the pathogenesis of multiple ocular diseases, including diabetic retinopathy, acute glaucoma and retinopathy of prematurity.^1^, ^2^ Moreover, retinal I/R injury developed vision loss and even blindness, owing to eternal damage to the retina, particularly retinal neurons.^3^, ^4^, ^5^ Ischaemia blocked blood flow to retina, causing the transient deficiency of oxygen and other physiological nutrients, such as adenosine triphosphate.^6^ The following reperfusion further aggravated the tissue damage through producing reactive oxygen species (ROS) and pro‐inflammatory mediators, which rendered oxidative stress and inflammation.^7^, ^8^ Therefore, the development of antioxidative and anti‐inflammatory treatments was gradually accepted as the main therapeutic strategies for retinal I/R injury.^9^

Carboxymethyl cellulose consisted of water‐soluble anionic polysaccharide and semisynthetic derivative of cellulose.^10^ It possessed a broad range of practical applications, such as pharmaceuticals,^11^ drug delivery^12^ and wound dressing,^13^ due to their high water content, biodegradability and biocompatibility. Interestingly, carboxymethyl cellulose, as functional medical materials, has been found to be able to reduce the high level of ROS.^14^, ^15^ However, this antioxidative capability of carboxymethyl cellulose against ROS was rather limited.^16^ An improving approach was modifying the cellulose with a stronger antioxidative group. Collagen peptide was known for its abundant physiological functions, such as chemotaxis, platelet aggregation, suppressing osteoclast differentiation and, in particular, protecting against oxidative free radical.^17^, ^18^, ^19^ Thus, the functionalization of carboxymethyl cellulose with collagen was expected to effectively improve its antioxidative capacity.

IL‐10, a potent anti‐inflammatory cytokine, is known for its potent capacity that hampered inflammation and modulated pathogenesis of inflammatory syndromes.^20^ The IL‐10 has been found to play a critical protective role in multiple inflammatory diseases, such as inflammatory bowel disease, experimental allergic encephalomyelitis and atherosclerosis.^21^, ^22^, ^23^

In this study, we prepared carboxymethyl cellulose grafted collagen peptides and investigated its antioxidative property in vitro against H_2_O_2_‐simulated oxidative stress environment. Then, IL‐10‐loaded antioxidative gels were delivered into the eyes of rats after retinal I/R injury for practical attempts. In addition, we preliminarily studied the underlying mechanism of enhanced restoration of retina from apoptosis with IL‐10‐loaded antioxidative gels by investigating the level of antioxidant enzyme and the mRNA expression of inflammatory cytokines.

## 2 MATERIALS AND METHODS

### 2.1 Materials

The commercial collagen peptide (*M*_w_ = 800) was purchased from Huashun Biological Technology Co. Ltd., China. The other chemicals at least analytical purity, including N‐Hydroxy sulfosuccinimide (NHS), 2‐(N‐morpholino) ethanesulfonic acid (MES) and 1‐ethyl‐(dimethylaminopropyl) carbodiimide (EDC), were purchased from Sinopharm Chemical Reagent Co., Ltd., China. Interleukin‐10 (IL‐10) and sodium carboxymethyl cellulose (*M*_w_ = 7.8 × 10^3^) were purchased from Sigma‐Aldrich. The culture medium was renewed every 48 hours when the cells were grown up to 80%‐90% confluence.

### 2.2 Preparation of carboxymethyl cellulose grafted collagen peptide

The synthetic approach was based on the previous method.^24^ Typically, first, carboxymethyl cellulose was first dissolved with magnetic stirring, followed by NHS‐EDC coupling reaction (EDC:CMC, 1:2). Then, after reacting for 1 hour, collagen peptide with the mass ratio of 5:3 to CMC was added into the mixture and the grafting reaction was undertaken in MES buffer (pH 6) for 20 hours at 55°C with a constant vigorous stirring. The final products, carboxymethyl cellulose grafted collagen peptide (CMCC), were lyophilized and stored before dialysis for 3 days.

### 2.3 Isolation and culture, viability and identification of retina endothelial cells

Three‐week‐old male Sprague‐Dawley (SD) rats were applied to the isolation of primary retina endothelial cells (rRECs), according to previous protocol.^25^ Typically, rats eyes were first enucleated and hemisected. The retinas were separated with a dissecting microscope, immersed in HBSS in the presence of penicillin/streptomycin (Sigma) and rinsed. A total of twenty‐four retinas were undertaken digestion with 0.5% collagenase type I (Worthington, NJ) in serum‐free DMEM for 45 minutes at 37°C. Then, endothelial cell‐specified medium containing 1% ECM (5% foetal bovine serum, 100 mg/mL streptomycin and 100 U/mL penicillin ScienCell Research Laboratories, CA) was supplemented. The obtained cells undertook filtering through 70‐ and 40‐μm nylon mesh (Becton, USA), successively, and were redispersed in ECM followed by incubating with goat antimouse magnetic beads pre‐coated via mouse anti‐rat PECAM‐1. After thoroughly washing with HBSS for 6 times and resuspending in ECM, the beads contained solution was then seeded on 6‐well plates pre‐treated with human fibronectin (Thermo Fisher Scientific, USA).

The viability of the resultant cells underwent propidium iodide (PI) evaluation for viability by flow cytometry, according to previously reported method.^26^ Briefly, after incubation with PI (100 ng/mL) in phosphate‐buffered saline (PBS) for 1 minute, cells were centrifuged to remove free PI and then resuspended in PBS containing 2% FBS for analysis using a BD LSR II flow cytometer (BD Biosciences, USA). Viable cells (PI cells) were calculated by flow cytometry at a constant flow rate. rRECs were confirmed by a positive immunofluorescence staining for endothelial cells markers: factor VIII‐related antigen. Briefly, cells, grown in cell slides of the 24‐well plates, were fixed with 4% paraformaldehyde for 10 minutes. After rinsing with PBS, they were blocked with 5% normal goat serum in PBS for 30 minutes and incubated with rabbit anti‐factor VIII‐related antigen polyclonal antibody (Santa Cruz, CA, USA) at 4°C overnight. Cells then were incubated with rhodamine‐conjugated anti‐rabbit secondary antibody at 37°C for 30 minutes. Afterwards, cells were washed with PBS 3 times and were staining with 4'6‐diamidino‐2‐phenylindole (DAPI, Sigma) for 5 minutes. The resulting samples were examined using a Olympus FV1000 confocal laser scanning microscope.

### 2.4 Cell viability assessment of rRECs

Cell viability was evaluated via methylthiazolyldiphenyl‐tetrazolium bromide (MTT) method (Sigma, USA) assay. The rRECs were cultivated in the medium containing different concentrations of H_2_O_2_ or μg/mL CMC. After 12‐hours incubation, the treated cells were rinsed and supplemented with 100 μL of DMEM containing MTT (20 μL in PBS, 5 mg/mL) for 4 hours. Subsequently, after removal of supernatant, 150 μL of DMSO was added and the absorbance of the solution in each well was recorded using a multimode microplate reader. Eight replicates were prepared for each treatment group.

### 2.5 Determination of intracellular reactive oxygen species

The intracellular ROSs were analysed using dihydroethidium (DHE) according to the previous protocol. Typically, rRECs were added with 1 mM DHE, incubating at 37°C for 25 minutes. The suspension was gently washed with PBS buffers for 3 times. The stained cells were then harvested and underwent FACScan flow cytometer (BD Biosciences) analysis.

### 2.6 Terminal deoxynucleotidyl transferase UTP nick end labelling (TUNEL) assay

TUNEL assay kits were purchased from Beyotime Biotechnology, China, and were performed as described in the manufacturer's protocols. Typically, the air‐dried rRECs were fixed in 4% paraformaldehyde and permeabilized with 0.1% Triton X‐100 containing 0.1% sodium citrate. Then, these cells were darkly incubated with TUNEL reagent. After washing, the cells were casted onto glass slides and immersed with a DAPI‐containing antifade mounting medium. TUNEL staining (green) images were attained through the Flouview‐FV300 Laser Scanning Confocal system (Olympus, Japan).

### 2.7 Western blotting assay

The proteins of lysed rRECs and retina were analysed using BCA^TM^ Protein Assay Kit (Pierce, USA), according to the manufacturer's protocol. Briefly, protein sample was first separated with SDS‐PAGE approach. The discrete protein strips were then electrophoretically transferred to nitrocellulose membranes, followed by incubating with primary antibodies against cleaved caspase 3 (Csp3), Bax, Bcl‐2, TNF‐α, IL‐1β, iNOS, ICAM‐1 and MCP‐1 overnight, respectively, and then the corresponding secondary antibodies marked with HRP for 1 hour. β‐Action and glyceraldehyde‐3‐phosphate dehydrogenase (GAPDH) served as internal reference for these cytokines. The corresponding antibodies were purchased from Cell Signal Technology, USA.

### 2.8 IL‐10 loading and releasing assay in vitro

The IL‐10 loading CMCC (IL‐10@CMCC) was obtained by soaking CMCC in saturated IL‐10 solution overnight at 4°C. The releasing assay of IL‐10@CMCC was performed using ELISA to detect the released IL‐10. Briefly, aliquots of the IL‐10@CMCC were separately dispersed and standing in 5 mL 1 × PBS (pH7.4). Subsequently, the supernatants were collected and analysed at different days. Each sample was repeated 3 times.

### 2.9 Retinal ischaemia/reperfusion injury

Sprague‐Dawley rats with similar weights were anesthetized using sodium pentobarbital and then received surgery, performed as described previously.^27^ Briefly, the anterior chambers of rat eyes were cannulated utilizing a 27‐gauge infusion needle connected with a normal saline reservoir. The intraocular pressure was then tuned up to 110 mm Hg for 1 hours. The retinal ischaemia/reperfusion was verified through examining the fundus whitening and the retinal blood flow restoration.

### 2.10 Injection of IL‐10@CMCC into vitreous

The injection procedures followed a previous protocol with modification.^28^ Briefly, after anaesthesia with ketamine (50 mg/kg) and phenergan (25 mg/kg), both pupils of rat eyes were dilated using 1% atropine and 1% compound tropicamide eye drops (Zhongshan Ophthalmic Center, China). 1% Tetracaine eye drops (Zhejiang Jiuxu Pharmaceutical Co., Ltd., China) was used for topical anaesthesia followed by the puncture of anterior chamber. Aqueous humour was aspirated to avoid the increase in intraocular tension, while eyeballs were immobilized with toothed forceps. Under surgical microscope, No. 4.5 needle (outlet: 0.46 mm, inlet: 0.25 mm) was inserted from pars plana and directed towards the midvitreous cavity. Subsequently, CMCC or IL‐10@CMCC in PBS was injected. The conjunctival sac was then casted with chlortetracycline eye ointment. After injection, rats underwent antibiotic eye drop for a week.

### 2.11 Measurement of retinal thickness

The retinal thickness of rats was measured as previously described.^29^ In brief, the rats were first anaesthetized at day 21, followed by the enucleation of their eyes. The eyecups then successively underwent the 2‐hours fixation in 4% paraformaldehyde, the 12‐hours immersion in 30% sucrose solution, and the final embedment in OCT media (Sakura Finetek, USA), after removing cornea, lens and vitreous. 0.5 mm of retinal sections were cut off along the vertical meridian of eye and across the optic nerve head. The cut‐off sections were stained with haematoxylin and eosin (H&E) and imaged via the light microscope. The retinal thickness measurements were taken at every 250 μm in a range of 800‐1200 μm centralized at the optic nerve head.

### 2.12 Immunofluorescence staining of Csp3

Typically, the cut‐off retinal sections received fixing with 4% paraformaldehyde for 10 minutes, permeabilizing with 0.1% Triton X‐100 for 15 minutes and blocking with 5% normal goat serum for 60 minutes. rRECs were then incubated with Csp3 antibody (1/200 diluted) overnight and secondary antibody modified with Alexa Fluor 568 for 1 hour (1/500 diluted, Molecular Probes, USA). DAPI staining was used as reference. Images were also attained via the Flouview‐FV300 Laser Scanning Confocal system.

### 2.13 Inflammation level assessment via quantitative polymerase chain reaction (PCR)

The extraction of total RNA from the retina was performed using TRIzol reagent (Invitrogen‐Life, USA). Then, 1 μg of total RNA was annealed with 300 ng of oligo (dT) (Promega, USA) for 5 minutes at 65°C, followed by reversely transcribing to cDNA utilizing 80U Moloney murine leukaemia virus reverse transcriptase (Gibco‐life, USA) at 37°C for 1 hour. The obtained cDNA was applied to real‐time qPCR using iTaq Universal SYBR Green Supermix (Bio‐Rad) using a CFX96 Touch Real‐Time PCR Detection System (Bio‐Rad). The following primers were used for amplification reaction: TNF‐α: forward primer 5’‐GCA TGA TCC GAG ATG TGG AA‐3’, reverse primer 5’‐ACG AGC GGG AAT GAG AAG AG‐3’; IL‐1β: forward primer 5’‐GGA GAA GCT GTG GAC GCT A‐3’, reverse primer 5’‐GCT GATGTA CCA GTT GGG GA‐3’; iNOS: forward primer 5’‐ TAT CTG CAG ACA CAT ACT TTA CGC‐3’, reverse primer 5’‐ TCC TGG AAC CAC TCG TAC TTG‐3’; ICAM‐1: forward primer 5’‐TCC TAA AAT GAC CTG CAG ACG‐3’, reverse primer 5’‐AGT TTT ATG GCC TCC TCC TGA‐3’; MCP‐1: forward primer 5’‐GCT CAT AGC AGC CAC CTT CAT TC‐3’, reverse primer 5’‐GTC TTC GGA GTT TGG GTT TGC‐3’.

### 2.14 Measurement of reactive oxygen species in retina

The chemilumigenic probes, luminol (5‐amino‐2,3‐dihydro‐1,4‐phthalazinedione, Sigma) and lucigenin (bis‐N‐methylacridiniumnitrate, Sigma), were used to assess the ROS level in rat retina.^30^ Briefly, retinas were isolated at days 3, 7 and 14 after euthanasia and then balanced with 1 × PBS. Samples were then placed into vials containing PBS‐HEPES buffer (pH 7.2). The level of ROS was analysed by supplementing lucigenin and luminol with a final concentration of 0.2 mM. The luminescent intensity was recorded at every 1 minutes through luminescence reader (BioTek, USA).

### 2.15 Measurements of the activities of antioxidant enzymes in retina

Colorimetric methods were aplicated to evaluate superoxide dismutase (SOD) and catalase (CAT) activities using kits (Cayman Chemical, USA) in the retina at day 3, 7 and 14. Assays were conducted according to the manufacturer's protocols.

### 2.16 Measurements of glutathione levels in retina

Glutathione (GSH) ELISA kits (Cayman Chemical, USA) were used to determine the concentration of GSH. The absorbance of the dithionitrobenzoic acid (DTNB) method at 412 nm at days 3, 7 and 14 was recorded and then calculated to the expression of GSH level as μmol/mg protein.

### 2.17 Statistical analysis

Digital data were expressed as mean ± SD. Statistical significance was denoted as *P* value < .05. The analysis within groups employed one‐way ANOVAs followed by Tukey's post hoc test for multiple pairwise tests.

## 3 RESULTS

### 3.1 rRECs viability and identification

**Figure 1:**
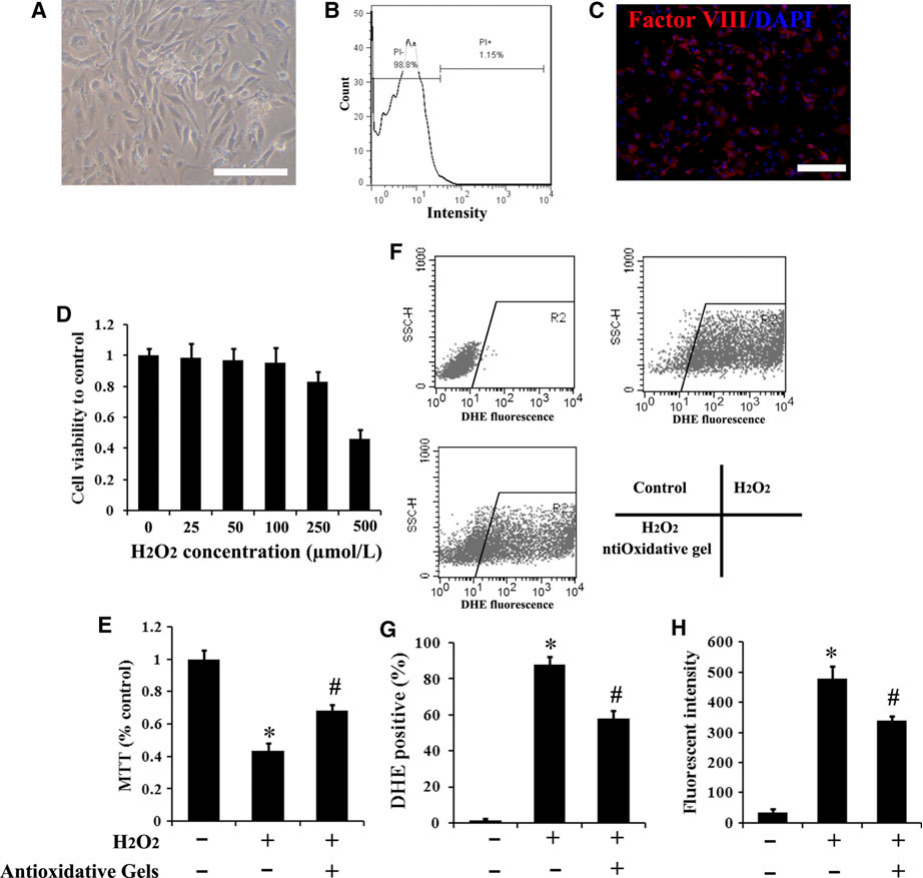
Characterization of rRECs and the effects of antioxidative gels, CMCC, on their intracellular ROS levels and apoptosis. A, Morphological image of isolated rRECs was attained by the inverted microscopy. Bar scale = 100 μm. B, Flow cytometry of rREC stained by PI. C, Immunofluorescence staining with antibodies against factor VIII‐related antigen showed positive staining in rRECs. Bar scale = 100 μm. D, The cell viability was dose‐dependently related to H_2_O_2_. E, The H_2_O_2_‐mediated decrease in cell viability was attenuated by add CMCC. F, Intracellular ROS levels in rRECs were analysed by flow cytometry. G and H, The quantitative detection of DHE staining‐positive rRECs and fluorescence intensity of DHE. (n = 5). * *P* < .05 compared with control; # *P* < .05 compared with H_2_O_2_ group

As can be seen in Figure 1A, the morphology of the isolated rRECs was fusiform. The PI evaluation showed that viable cells (PI‐) reached 98.8%, indicating desirable isolation efficiency (Figure 1B). Furthermore, the obtained cells were immunofluorescence stained with the specific biomarker of rRECs (factor VIII) and DAPI for cell nucleus. As shown in Figure 1C, the almost complete overlapping of the 2 markers indicated the high purity of rRECs.

### 3.2 CMCC protect rRECs against H_2_O_2_‐induced oxidative stress

The introduction of H_2_O_2_ into rRECs culture medium was used to establish in vitro oxidative stress model and assess whether CMCC was able to strengthen the viability of rRECs under the condition. As shown in Figure 1D, the viability of rRECs decreased when the H_2_O_2_ concentration elevated up to 250 μmol/L. When the concentration of H_2_O_2_ reached 500 μmol/L, only around half of the cells can survive and this level of H_2_O_2_ was thus chosen in the following experiments. The CMCC was known for its antioxidative capability. As predicted, the 500 μmol/L H_2_O_2_‐induced decrease in rRECs viability can be considerably offset by the addition of this antioxidative gels (Figure 1E). Furthermore, as shown in Figure 1F‐H, normal rRECs possessed a relatively low ROS level. Hence, few DHC‐positive cells and weak fluorescent intensity were detected. However, DHE response dramatically increased after introducing H_2_O_2_. This augment generated by H_2_O_2_ could be significantly attenuated (*P* < .05) by adding antioxidative gels.

**Figure 2:**
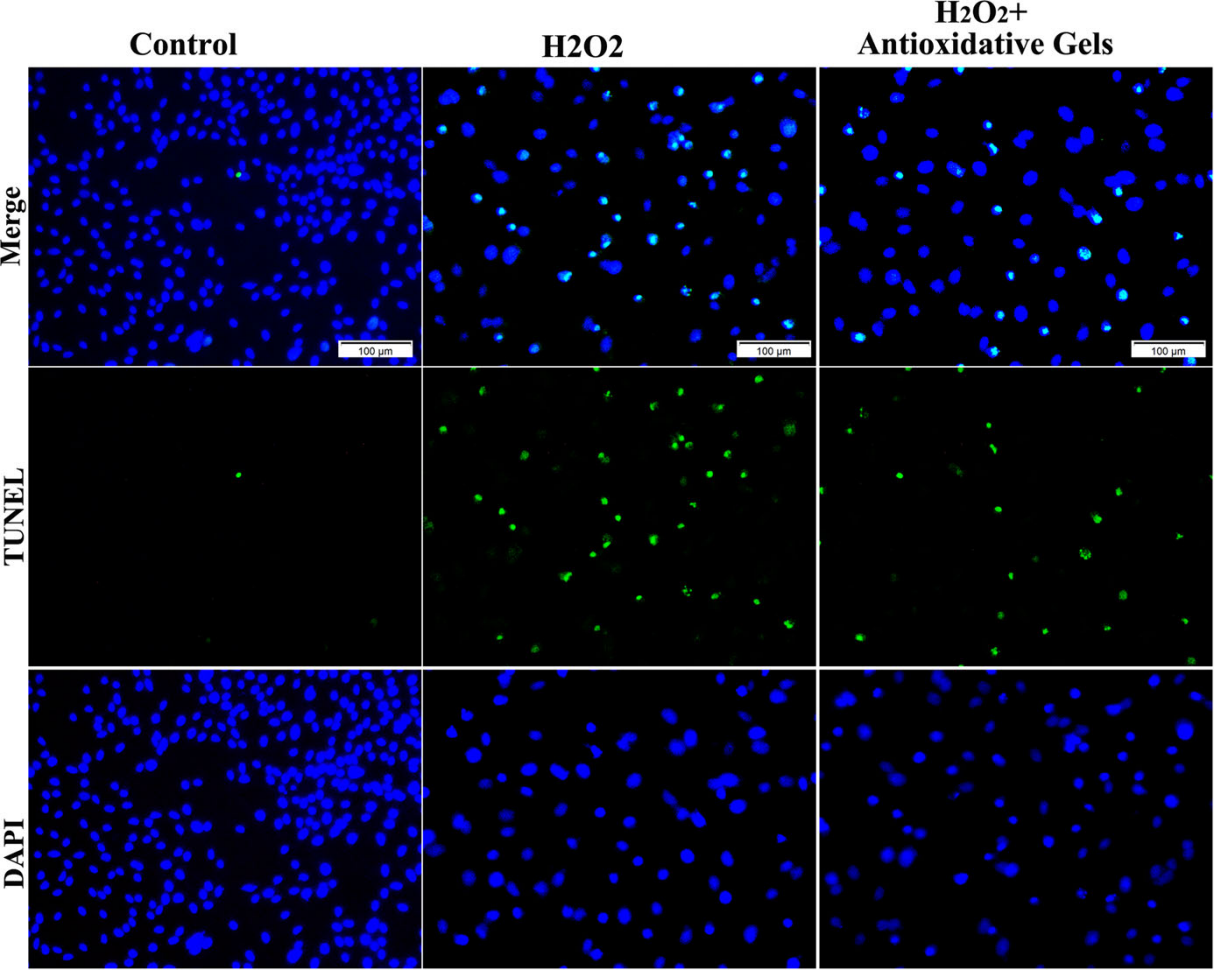
Representative TUNEL images of rRECs. The rRECs were pre‐treated with or without H_2_O_2_ and antioxidative gels. The blue was nucleus staining with DAPI and the green was TUNEL staining, representing apoptotic rRECs. Three groups. Bar scale = 100 μm

**Figure 3:**
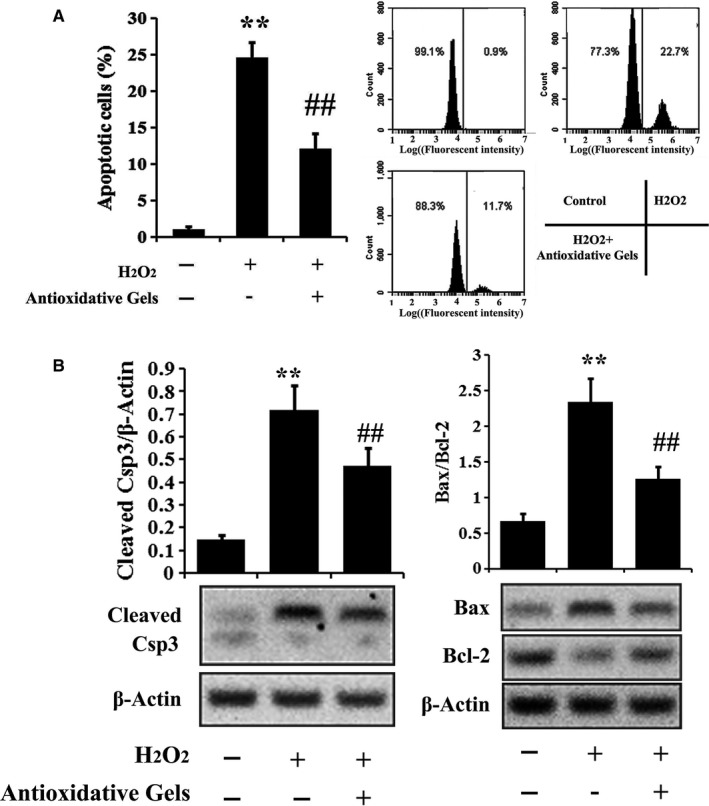
Assessment of apoptosis‐related proteins in rRECs. A, Apoptosis of rRECs treated with or without H_2_O_2_ or following antioxidative gels was analysed by flow cytometry. B, The levels of apoptotic proteins in H_2_O_2‐_treated rRECs were analysed by Western blotting (n = 5). ** *P* < .01 compared with control; ## *P* < .01 compared with H_2_O_2_ group

The typical TUNEL staining images (Figure 2) were in agreement with the flow cytometry measurements. TUNEL‐positive cells were much less in the control group than that in the H_2_O_2_‐treated one, while the increased number of TUNEL‐positive cells by oxidative stress was reduced via the introduction of CMCC. The following quantification results of TUNEL by flow cytometry (Figure 3A) clearly indicated that apoptosis in the control group was significantly lower than H_2_O_2_‐treated group and this change could be considerably ameliorated by supplementing antioxidative gels.

### 3.3 Assessment of apoptotic proteins

The investigation of apoptotic proteins expression in rRECs further validated the underlying mechanism. As shown in Figure 3B, H_2_O_2_‐induced oxidative stress significantly up‐regulated pro‐apoptotic protein, Csp3 and the ratio of Bax to Bcl‐2. This modulation in rRECs was then significantly suppressed when the antioxidative gels, CCPCC, were introduced.

### 3.4 In vitro assay of IL‐10 releasing

**Figure 4:**
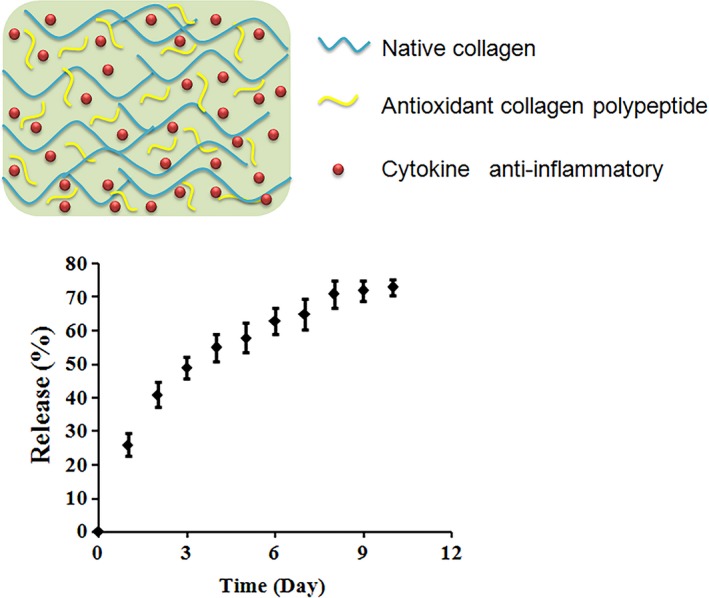
IL‐10 releasing measurement in vitro. Upper panel) The schematic image of IL‐10@CMCC structure. Bottom panel) the releasing progress was recorded at 1‐day interval and up to 10 d

The released IL‐10 in supernatant was measured at days 0, 1, 2, 3, 4, 5, 6, 7, 8, 9 and 10, respectively (Figure 4). It can be seen that about 25% amount of drugs were released at the first day. With time, the releasing speed was correspondingly decreased. At day 10, only 2% increase was detected, compared with the releasing amount at day 9, indicating the relative retention of loaded IL‐10 in antioxidative gels.

### 3.5 Effects of IL‐10@CMCC on retinal histology after I/R injury

**Figure 5:**
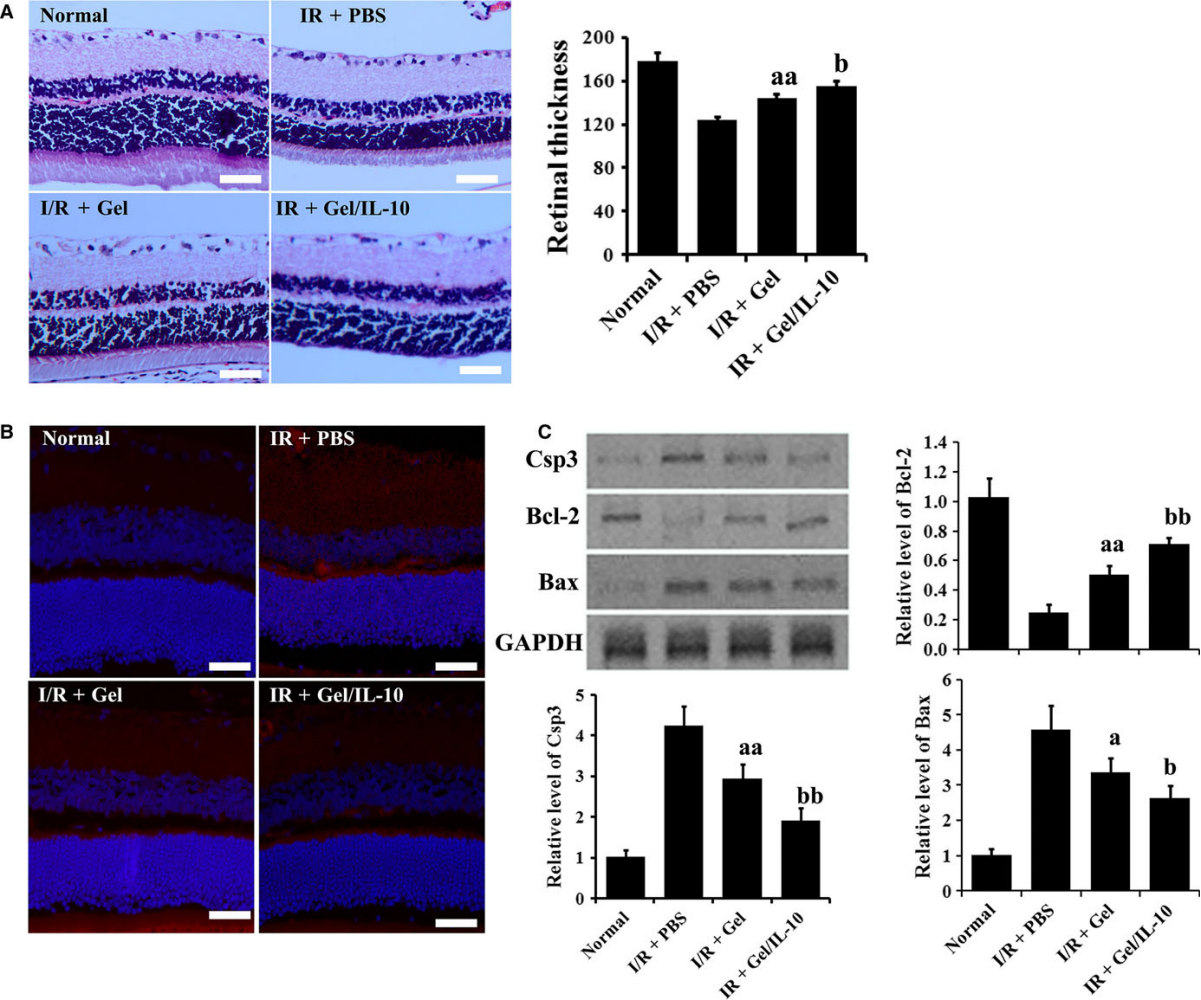
Effects of IL‐10@CMCC on retinal histology at 21 d post‐I/R injury. A, Representative retinal section images of control, I/R + PBS, I/R + Gel or I/R + Gel/IL‐10 group were attained by H&E staining and the corresponding quantitative total retinal thickness (from the inner limiting membrane to the pigment epithelium). B, Representative immunofluorescence images specified for Csp3 in the 4 groups. C, Western blotting analysis of apoptotic proteins of rRECs, for example Csp3, Bcl‐2 and Bax, in the 4 groups. n = 5, bar scale = 50 μm, a *P* < .05 compared with I/R + PBS group; aa *P* < .01 compared with I/R + PBS group; b *P* < .05 compared with I/R + Gel group; bb *P* < .01 compared with I/R + Gel group

As shown in Figure 5A, retinal I/R injury obviously caused the decrease in retinal thickness, compared with normal group at day 21. Marked increase in retinal thickness was found in the I/R + Gel group and this restoration was enhanced in the I/R + Gel/IL‐10 group, compared with the I/R group. The data of bar plotting clearly supported the observation through H&E staining. In addition, the thicknesses of inner plexiform layer (IPL), inner nuclear layer (INL) and outer nuclear layer (ONL) were generally reduced by the treatment of I/R (Table 1). The introduction of antioxidative gels significantly attenuated the thinness. The beneficial effect of gels could be further amplified by the loading of IL‐10.

**Table 1:** The thickness of the inner plexiform layer (IPL), the inner nuclear layer (INL) and the outer nuclear layers (ONL) in 4 groups

	Normal	I/R + PBS	I/R + Gel	IR + Gel/IL‐10
IPL (μm)	68.2 ± 3.7	42.4 ± 3.0	51.4 ± 2.3^a^	57 ± 3.4^b^
INL (μm)	40.4 ± 2.4	26.7 ± 2.0	33.0 ± 1.7^a^	36.6 ± 2.0
ONL (μm)	57.7 ± 1.3	41 ± 2.6	48.8 ± 1.1^a^	52.4 ± 1.9^b^

n = 5.

a *P* < .05 compared with I/R + PBS group.

b *P* < .05 compared with I/R + Gel group.

The immunofluorescence staining of Csp3 revealed the apoptosis in retina (Figure 5B). The red dots were notably emerged in I/R group, compared with the normal one. However, the number and distribution of red stains were decreased in I/R + Gel group. Moreover, a further decrease was found in the I/R + Gel/IL‐10 group. Furthermore, as shown in Figure 5C, Western blotting of apoptosis‐related proteins clearly showed that the introduction of gels significantly elevated the expression of anti‐apoptotic protein, for example Bcl‐2 and lowered that of apoptotic proteins, for example Csp3 and Bax. Such modulation was further enhanced by loading IL‐10, indicating the protective effect of antioxidative gels and IL‐10 against the retinal apoptosis induced by I/R.

### 3.6 Effects of IL‐10@CMCC on the expression of inflammatory cytokine

**Figure 6:**
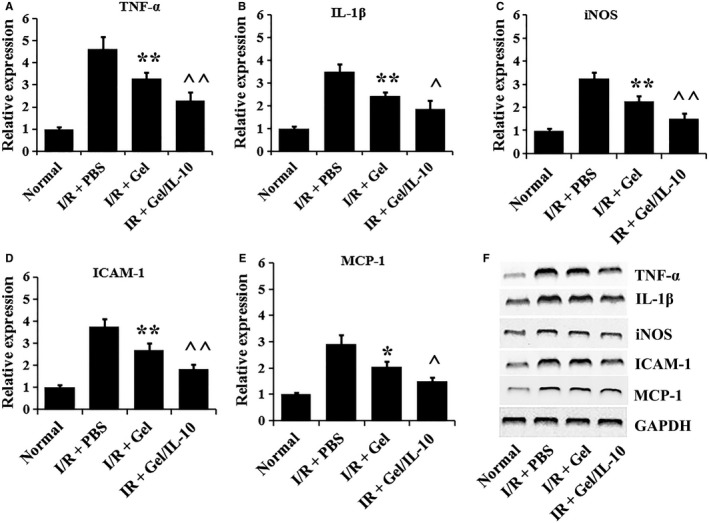
Effects of IL‐10@CMCC on the expression of inflammatory mediators in retinal cells after I/R injury. At day 3, the mRNA expression of inflammatory mediators, for example A, TNF‐α, B, IL‐1β, C, iNOS, D, ICAM‐1 and E, MCP‐1 in retinas of control, I/R + PBS, I/R + Gel or I/R + Gel + IL‐10 group, was investigated via qPCR based on 2^‐Ct (n = 5). F) Western blotting images of the corresponding mediators in the 4 groups. * *P* < .05 compared with I/R + PBS group; ** *P* < .01 compared with I/R + PBS group; ^ *P* < .05 compared with I/R + Gel group; ^^ *P* < .01 compared with I/R + Gel group

At day 3, as shown in Figure 6A‐E, the mRNA expression levels of TNF‐α, IL‐1β, iNOS, ICAM‐1 and MCP‐1 were much higher in I/R‐injured group than the normal rats. In I/R + Gel, the expression of TNF‐α, IL‐1β, iNOS, ICAM‐1 and MCP‐1 significantly reduced, compared with that in I/R group (*P* < .05). Moreover, the mRNA levels of TNF‐α, IL‐1β, iNOS, ICAM‐1 and MCP‐1 were significantly less in the IL‐10 involved group than in the only CMCC containing group (*P* < .05). The further Western blotting results (Figure 6F) provided a supportive evidence for the effects of IL‐10@CMCC on the expression of the inflammatory cytokines.

### 3.7 Effects of IL‐10@CMCC on the level of ROS and the activity of anti‐oxidases

**Figure 7:**
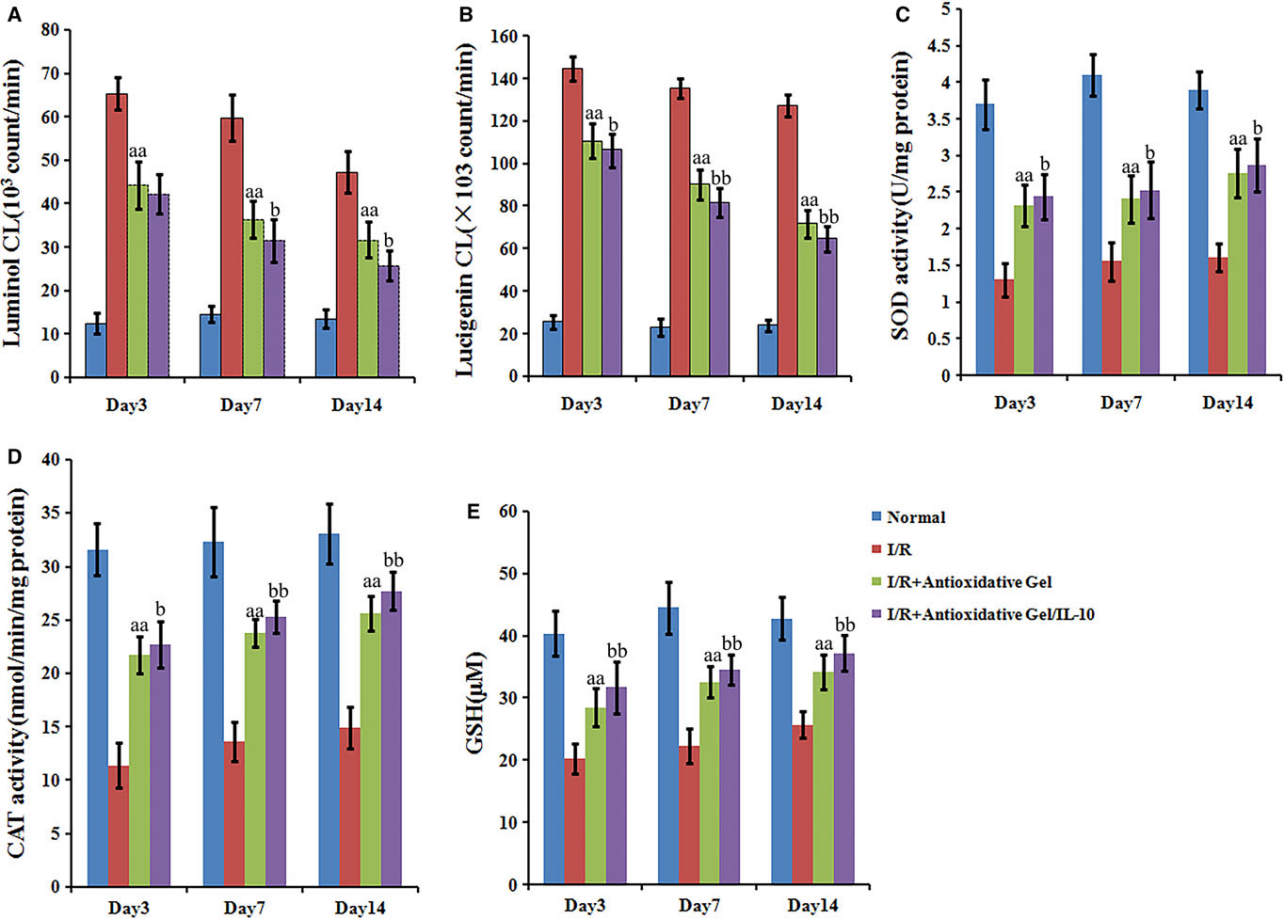
Effects of IL‐10@CMCC on retinal ROS and anti‐oxidase activity post‐I/R injury. A, Luminol‐ and (B) lucigenin‐enhanced chemiluminescence as well as the activity of (C) SOD and (D) CAT enzymes as well as E) GSH levels in retinas of the control, I/R + PBS, I/R + CMCC or I/R + IL‐10@CMCC group were investigated at days 3, 7 and 14, respectively (n = 5). aa *P* < .01 compared with I/R + PBS group; b *P* < .05 compared with I/R + Gel group; bb *P* < .01 compared with I/R + Gel group

The changes in oxidative states in retinas were finally assessed by evaluating the level of ROS and the activity of antioxidant enzymes. Lucigenin‐ and luminol‐enhanced chemiluminescence (CL) methods revealed the in vivo ROS level at days 3, 7 and 14 respectively. As shown in Figure 7, I/R injury could significantly trigger the CL in retina. The administration of antioxidative gels and IL‐10 remarkably decreased the luminol‐ and lucigenin‐enhanced CL signal over 2 weeks, compared with I/R group. Conversely, the activity of SOD and CAT as well as the level of GSH in retina was found significantly reduced in I/R group, compared with those in normal group, as shown in Figure 7C,D. Such significant decrease in the activity of SOD and CAT enzymes and GSH level induced by I/R injury was considerably inhibited by the supplementation with antioxidative gels, CMCC, particularly loaded with IL‐10 at days 3, 7 and 14.

## 4 DISCUSSION

Oxidative stress and inflammation were reported to play key roles in retinal damage caused by I/R injury.^31^, ^32^ Hence, drugs or treatments capable of attenuating ROS level or/and inflammation in retina are considered as efficacious and main therapies for retinal I/R injury.^31^ H_2_O_2_ was regarded as one of the most important species in ROS, due to its stability and key status in ROS‐related metabolism.^33^, ^34^ Thus, in vitro H_2_O_2_ tests were gradually accepted as featured tools for the study of ROS or oxidative stress.^35^, ^36^ Here, H_2_O_2_ was employed to simulate in vitro oxidative stress microenvironment and rRECs were selected as model cells due to its physiological function of retinal vascularization, which is essential for structural and functional recovery of the retina.^37^

Our study was aimed at the establishment of a desirable drug slow‐release system for the retinal treatment of I/R injury. The antioxidative gel carriers, CMCC, were found to significantly decrease intracellular ROS level and thus apoptosis in rRECs. The following analysis of the apoptotic cytokines offered us evidence in molecular level for the CMCC‐based protection on rRECs. The accumulation of intracellular ROS could inhibit the Akt‐signal pathway,^38^ followed by down‐regulating survival signals, including Bcl‐2 and activating pro‐apoptotic factors, such as Bax and Csp3.^39^ Additionally, the high concentration of ROS was conducive to triggering the opening of the mitochondria permeability transition gates, thus leading to the release of apoptosis‐activating cytokines.^40^ Moreover, the expression of pro‐apoptotic protein of mitochondria membrane, Bax, was enhanced and anti‐apoptotic protein, Bcl‐2, was, correspondingly, blocked. The continuing apoptotic response would ultimately activate the late‐stage apoptotic protein, Csp3.^41^, ^42^

The therapeutic efficiency of this antioxidative gel cargo could be further enhanced by loading drugs. IL‐10 was selected for its anti‐inflammation drug potentials.^43^ Previous studies have already utilized IL‐10 to mitigate inflammatory response to I/R injury.^44^, ^45^ For instance, the up‐regulation of IL‐10 through pre‐conditioning with CpG‐oligonucleotides could mitigate the myocardial I/R injury.^46^ In this study, we demonstrated that IL‐10 load enhanced the protective effect of CMCC on retinal cells against I/R injury. The restoration of retinal morphology and the decrease in apoptosis confirmed the therapeutic effect of IL‐10. In addition, the IL‐10@CMCC delivery system synergistically mitigated retinal oxidative stress damage and suppressed the expression of inflammatory cytokines (TNF‐α, IL‐1β, iNOS, ICAM‐1 and MCP‐1). The pathogenesis of retinal I/R injury was found to up‐regulate the expression of multiple inflammatory mediators, including TNF‐α, IL‐1β, iNOS, ICAM‐1 and MCP‐1, which was in accordance with previous reports.^3^ TNF‐α played a pivotal role in regulating neuron death caused by retinal I/R injury.^47^, ^48^ IL‐1β was involved in the degeneration of inner retinal compositions owe to I/R injury.^49^ The iNOS‐triggered delayed neuronal cell death after ischaemia mediated the production of nitric oxide (NO),^50^ which would finally develop into inflammatory oxidants.^51^ ICAM‐1 directly activated inflammatory responses to the blood vessel wall through stimulating endothelial cell and generating atherosclerotic plaque.^52^ MCP‐1 contributed to the combination between monocytes/macrophages and target tissues.^53^ In this study, we clearly demonstrated that the drug‐carrying platform, antioxidant gels loading with IL‐10, could inhibit the expression of TNF‐α, IL‐1β, iNOS, ICAM‐1 and MCP‐1. Our results complied with those of previous studies,^54^, ^55^ demonstrating that the gels and IL‐10 synergistically displayed anti‐inflammatory activities through the expression inhibition of various inflammatory mediators.

Afterwards, another important factor to induce apoptosis after I/R, in vivo oxidative stress, was investigated. Previous studies have confirmed that I/R injury produced a myriads of ROS, including H_2_O_2_, OH^**‐**^, hypochlorite, peroxynitrite and superoxide radicals, resulting in cellular damage and apoptosis.^3^, ^31^ Our data of in vivo ROS analysis were consistent with that of the previous report,^27^ indicating that I/R injury induced apoptosis in retina was accompanied by significant augment of ROS level. In addition, the decreases in oxidant scavenges, typically SOD, CAT and GSH, were found after I/R injury,^27^ which further weakened the capacity of the degradation of intracellular ROS. In our study, the IL‐10@CMCC hybrids were capable of ameliorating the augment of ROS level. After injecting these IL‐10‐loaded hydrogels, the expressive abnormity of ROS and oxidant scavenges was significantly offset, facilitating to recover retinal cells from apoptosis and ultimately retinal thickness as well as morphology. Albeit plausible results achieved in retinal treatment post‐I/R injury, there is still a long way to improve therapeutic effects of drug‐delivery system on the retina I/R injury. We hence need more endeavour to explore and optimize the in vivo performance of this slow‐releasing system in the future.

## 5 CONCLUSION

In summary, we here utilized H_2_O_2_ to simulate in vitro oxidative stress microenvironment and thus tested the performance of CMCC, the drug carrier, establishing the antioxidative and thus anti‐apoptotic features of this drug cargo. The next in vivo study demonstrated that I/R injury induced oxidative stress and inflammation could be potently mitigated by the treatment of these drug slow‐releasing systems. The reduction in apoptotic retinal cells and thus the raise of retinal thickness fully demonstrated the therapeutic effects of IL‐10@CMCC on retinal I/R injury.

## CONFLICT OF INTEREST

The authors declare that there is no conflict of interest regarding the publication of this paper.
